# Supplementary material for: High-Temperature and Drought Stress Effects on Growth, Yield and Nutritional Quality with Transpiration Response to Vapor Pressure Deficit in Lentil
Source: Plants (Basel). 2021 Dec 28;11(1):95. doi: 10.3390/plants11010095 (PMC8747359; doi:10.3390/plants11010095)
Supplement: Supplementary file 1 [file plants-11-00095-s001.zip › plants-1509024-supplementary.pdf]

Table S1. ANOVA analysis for 20 lines of under normal and late planting trials at Marchouch.

	PLH	EGV	DFF	D50F	D50P	DM	NFPP	NUPP	NTPP	GYP	HSW	CP	Zn	Fe
Gen (G)	**	**	**	**	**	**	*	*	*	*	**	**	**	**
Trt (T)	**	**	**	**	**	**	**	**	**	**	**	**	**	**
G*T	*	**	**	*	**	*	*	ns	*	*	ns	**	**	**
Error	2.7	2.4	0.9	3.3	2.7	1.2	859.3	19.2	868.8	1.3	0.1	0.2	15.4	16.1
CV	7.1	7.9	1.3	2.4	1.8	0.9	36.8	49.3	25.8	35.3	12.9	2.1	9.1	5.2
R^2	0.92	0.92	0.99	0.98	0.99	0.99	0.78	0.64	0.79	0.75	0.91	0.99	0.90	0.92

*, ** and ns indicate significant difference at 0.01 and 0.001, and ns denotes a non-significant. PLH, plant height; EGV, early growth vigor; DFF, days to first flowering; D50F, days to 50% flowering; D50P, days to 50% podding, DM, days to 95% maturity; NFPP, number of filled pods per plant; NUPP, number of unfilled pods per plant; NTPP, number of total pods per plant; GYP, grain yield per plant; HSW, hundred-seed weight; CP, crude protein; Zn, zinc content; Fe, iron content.

Table S2. Correlation between tested traits under normal conditions conducted at Marchouch.

	PLH	EGV	DFF	D50F	D50P	DM	NFPP	NUPP	NTPP	GYP	HSW	CP	Zn	Fe
**PLH**	1													
**EGV**	**.38***	1												
**DFF**	-.02	**-.46****	1											
**D50F**	-.10	**-.53****	**.70****	1										
**D50P**	-.15	**-.61****	**.49****	.30	1									
**D95M**	-.24	-.29	-.12	.01	.13	1								
**NFPP**	.25	.08	-.14	.03	-.23	.04	1							
**NUPP**	**-.40***	-.23	.09	-.06	.15	.01	-.28	1						
**NTPP**	.21	.05	-.13	.02	-.22	.04	**.99****	-.16	1					
**GYP**	.23	.16	-.15	.01	-.29	.02	**.97****	-.23	**.96****	1				
**HSW**	.24	**.62****	**-.53****	**-.58****	**-.48****	-.21	.07	-.14	.05	.12	1			
**CP**	-.02	-.03	.03	.10	-.10	-.31	.03	.09	.05	.01	-.13	1		
**Zn**	-.02	-.27	.04	-.14	.30	.22	-.03	.01	-.03	-.08	.02	**-.47****	1	
**Fe**	-.27	**-.61****	.19	.14	**.52****	**.38***	-.21	.19	-.19	-.28	**-.39***	**-.35***	**.68****	1

* and ** indicate significant difference at 0.05 and 0.01. PLH, plant height; EGV, early growth vigor; DFF, days to first flowering; D50F, days to 50% flowering; D50P, days to 50% podding, DM, days to 95% maturity; NFPP, number of filled pods per plant^{-1}; NUPP, number of unfilled pods per plant^{-1}; NTPP, number of total pods per plant^{-1}; GYP, grain yield per plant^{-1}; HSW, hundred-seed weight; CP, crude protein; Zn, zinc content; Fe, iron content.

Table S3. Correlation for late under combined temperature-drought stress (above) and temperature stress (below).

	PLH	EGV	DFF	D50F	D50P	DM	NFPP	NUPP	NTPP	GYP	HSW	CP	Zn	Fe
**PLH**	1	.40**	-.10	-.02	-.15	-.13	-.07	.06	-.06	-.03	.16	-.14	.09	.21
**EGV**	.18	1	-.43**	-.40**	-.39*	-.44**	-.23	-.21	-.27	-.11	.25	-.11	.12	.26
**DFF**	-.06	-.41**	1	.66**	.54**	.42**	.03	.09	.05	.02	.08	.17	-.28	-.17
**D50F**	-.17	-.47**	.81**	1	.77**	.63**	.07	-.02	.07	.03	.34*	.19	-.31*	-.11
**D50P**	-.07	-.52**	.68**	.79**	1	.74**	.16	.19	.19	.16	.30	.16	-.24	-.15
**DM**	-.05	-.21	.19	.32*	.40*	1	.32*	.11	.34*	.25	.12	.12	-.40*	-.19
**NFPP**	.01	.14	-.20	-.09	-.07	.08	1	-.06	.98**	.90**	-.11	.06	-.09	-.10
**NUPP**	.15	-.09	.05	.13	.17	.09	.05	1	.13	-.02	-.11	.15	-.09	-.24
**NTPP**	.03	.13	-.19	-.08	-.05	.09	.99**	.15	1	.89**	-.13	.09	-.11	-.15
**GYP**	-.04	-.02	-.15	.01	-.03	-.07	.88**	-.02	.86**	1	.02	.05	-.01	-.04
**HSW**	-.25	-.02	.53**	.38*	.25	-.22	-.13	-.08	-.14	-.04	1	.32*	-.14	.02
**CP**	.03	-.06	-.09	-.15	-.12	.36*	.28	-.03	.28	.12	-.31	1	-.45**	-.43**
**Zn**	.01	.12	.29	.29	.16	-.15	-.17	.24	-.14	-.12	.34*	-.36*	1	.48**
**Fe**	-.01	-.24	.25	.37*	.29	-.01	.26	.03	.26	.34*	.14	-.11	.46**	1

* and ** indicate significant difference at 0.05 and 0.01. PLH, plant height; EGV, early growth vigor; DFF, days to first flowering; D50F, days to 50% flowering; D50P, days to 50% podding, DM, days to 95% maturity; NFPP, number of filled pods per plant^{-1}; NUPP, number of unfilled pods per plant^{-1}; NTPP, number of total pods per plant^{-1}; GYP, grain yield per plant^{-1}; HSW, hundred-seed weight; CP, crude protein; Zn, zinc content; Fe, iron content.

Table S4. Contribution of traits to the three first PCA dimensions under normal conditions, temperature stress, and the combined temperature-drought stress.

Traits	Normal			Temperature stress			Temperature-drought stress		
	PC1	PC2	PC3	PC1	PC2	PC3	PC1	PC2	PC3
PLH	6.21	1.45	0.20	2.36	0.01	0.00	1.55	0.64	19.81
EGV	13.82	6.89	0.11	5.78	6.05	7.37	8.35	0.09	1.16
DFF	5.69	5.13	10.94	18.01	3.05	0.02	8.46	7.05	1.95
D50F	3.23	12.15	14.53	18.00	6.66	0.00	11.38	7.30	11.18
D50P	17.59	0.51	0.15	15.23	6.12	2.27	12.29	4.13	5.21
DM	4.87	0.83	8.63	1.14	5.89	24.59	16.56	0.48	2.11
NFPP	5.83	21.55	0.85	8.51	19.57	2.72	6.21	24.03	1.48
NUPP	5.39	2.66	0.31	0.07	1.31	0.10	7.30	22.50	0.46
NTPP	4.53	22.24	0.77	8.02	19.91	2.73	0.98	1.55	14.36
GYP	5.56	20.82	0.54	4.74	21.15	5.37	4.91	21.10	2.02
HSW	11.41	2.83	7.80	8.77	0.31	8.70	0.33	6.91	7.12
CP	0.46	0.17	18.38	2.79	3.52	17.92	5.66	3.40	8.83
Zn	2.80	0.97	25.43	5.79	0.21	18.69	7.65	0.73	6.88
Fe	12.60	1.79	11.36	3.22	7.55	9.63	8.36	0.08	17.43

PLH, plant height; EGV, early growth vigor; DFF, days to first flowering; D50F, days to 50% flowering; D50P, days to 50% podding, DM, days to 95% maturity; NFPP, number of filled pods per plant^{-1}; NUPP, number of unfilled pods per plant^{-1}; NTPP, number of total pods per plant^{-1}; GYP, grain yield per plant^{-1}; HSW, hundred-seed weight; CP, crude protein; Zn, zinc content; Fe, iron content.

Table S5. Analysis of variance for canopy temperature between normal conditions, temperature stress and the combined temperature-drought stress at Marchouch.

Source	df	Mean Square	F
TRT	9	58.11*	64.54
Gen	19	2.99*	3.33
Rep	1	1.50ns	1.67
TRT * Gen	171	.89ns	.99

* indicates significant difference at 0.001 of probability, and ns denotes non-significant

Table S6. Minimum, Maximum, Mean of canopy temperature under normal conditions, temperature stress and the combined temperature-drought stress at Marchouch.

	Temperature-drought stress				Temperature stress				Normal	
**Days**	**65**	**70**	**80**	**90**	**65**	**70**	**80**	**90**	**100**	**115**
Min	27.80	29.70	29.30	28.40	27.70	29.50	29.30	29.60	26.60	29.70
Max	31.30	33.60	33.90	34.00	31.30	33.10	32,40	33.80	32.70	33.40
Mean±SD	29.53e± 0.8	31.89ab ±0.8	31.64cd ±1.1	31.76abc ±1.2	29.20f± 0.8	31.67bcd ±0.9	31.50d± 0.8	31.96a± 0.8	29.10f± 1.4	31.78abc ±1.1
LSD	0.61	0.59	0.73	1.01	0.54	0.62	0.64	0.69	1.07	0.78
CV	2.50	2.16	2.73	4.22	2.17	2.31	2.47	2.57	4.78	2.93

Means followed by the same letters are not significantly different (p<0.05).

Table S7. Description of origin, IG number and classification of lentil genotypes used in the current study. MHT: Moderately heat tolerant; MDT: Moderately drought tolerant, HT: heat tolerant, HS: heat sensitive, DT: drought tolerant, DS: drought sensitive.

Genotype	IG number	Origin	Classification
ILL 3484	3484	Nepal	MT
Bakria	4605	Morocco	MT
ILL 5919	69528	Ethiopia	HS; DS
ILL 6075	70130	Pakistan	DT
ILL 6104	70159	Pakistan	HT
ILL 6338	71270	Pakistan	HT
ILL 6359	71291	Pakistan	MT
ILL 6362	71294	Pakistan	HDT
ILL 6363	71295	Pakistan	MT
ILL 7223	75942	Nepal	MT
ILL 7286	76005	Nepal	HT
ILL 7804	114892	Nepal	HDT
ILL 7813	114929	Nepal	HS; HDS
ILL 7814	114931	Nepal	HT; HDT
ILL 7819	114948	Nepal	MT
ILL 7820	114951	Nepal	HS; HDS
ILL 7833	115006	Nepal	HT
ILL 7835	115010	Nepal	HT; HDT
ILL 8025	117726	Pakistan	MT
ILL 8029	117734	Pakistan	HT

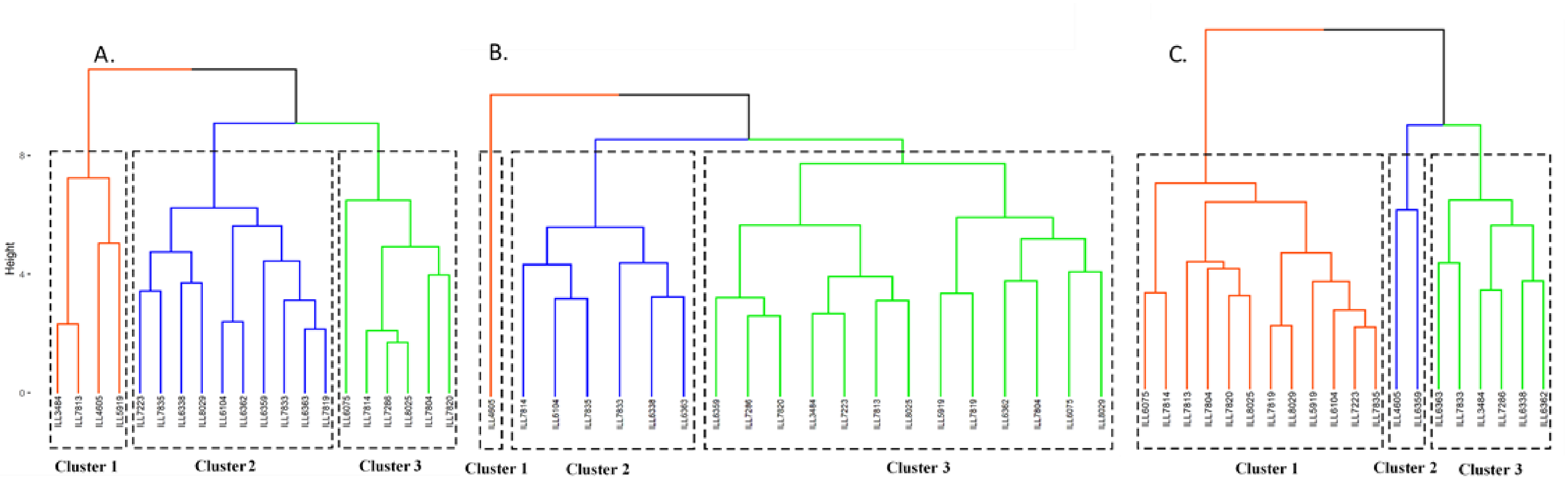

Figure S1. Dendrogram normal (a), temperature stress (b) and the combined temperature-drought stress (c) condition.
